# Synchronous gallbladder metastasis of renal cell carcinoma presenting as a gallbladder polyp: A case report

**DOI:** 10.1097/MD.0000000000024037

**Published:** 2021-01-22

**Authors:** Sung Hoon Cho, Young Seok Han, Ja Ryung Han, Hyung Jun Kwon, Seock Hwan Choi, Hyun Tae Kim, Man-Hoon Han, Jae Min Chun

**Affiliations:** a Department of Surgery; b Department of Urology; c Department of Pathology, Kyungpook National University Hospital, School of Medicine, Kyungpook National University, Daegu, Republic of Korea.

**Keywords:** gallbladder polyp, metastasis, renal cell carcinoma

## Abstract

**Rationale:**

Gallbladder polyps are common in the general population, but gallbladder metastasis of renal cell carcinoma (RCC) is very rare. In a patient with RCC diagnosed with a small gallbladder polyp that does not meet the traditional size criteria, the surgeon faces a dilemma of whether cholecystectomy should be performed given the possibility of metastasis.

**Patient concerns:**

A 55-year-old man who had received a left nephrectomy for RCC presented with a gallbladder polyp that was noted at the time of the nephrectomy. Imaging showed the maximum diameter of the polyp had increased from 5 mm to 24 mm in the 40 months after the initial diagnosis.

**Diagnosis:**

Pathological and immunohistology findings confirmed the gallbladder polyp as a metastasis of clear-cell RCC.

**Interventions:**

: We performed a laparoscopic cholecystectomy.

**Outcomes:**

Even though the synchronous solitary gallbladder metastasis was left untreated and a cholecystectomy was not performed over the 40 months, no metastasis occurred in other sites. The patient is free from disease 10 months after the cholecystectomy.

**Lessons:**

Solitary gallbladder metastasis of RCC may have more favorable outcomes than typical metastases. Although gallbladder metastasis of RCC occur rarely, it can occur, and any changes in gallbladder polyps in RCC patients should be managed under a strong suspicion of metastasis.

## 1 Introduction

Gallbladder polyps are sometimes observed in the clinical setting and have an estimated prevalence between 0.3% and 9.5%.^[1–3]^ The prevalence of gallbladder polyps is expected to increase because of extension of the average lifespan, changes in dietary patterns, improvements in imaging modalities, and health-screening programs.

Metastasis of renal cell carcinoma (RCC) is common and affects mainly the lung, bone, liver, adrenal glands, brain, and other kidney. Gallbladder metastases from RCC may also occur but are very rare.^[4,5]^ One challenge for the surgeon is the decision about whether to remove the gallbladder in a patient diagnosed with RCC with concurrent gallbladder polyps that do not meet the traditional indications for cholecystectomy.

Herein, we report a case involving a gallbladder polyp that was identified as a synchronous metastasis of RCC found 40 months after the nephrectomy.

## 2 Case report

**Figure 1:**
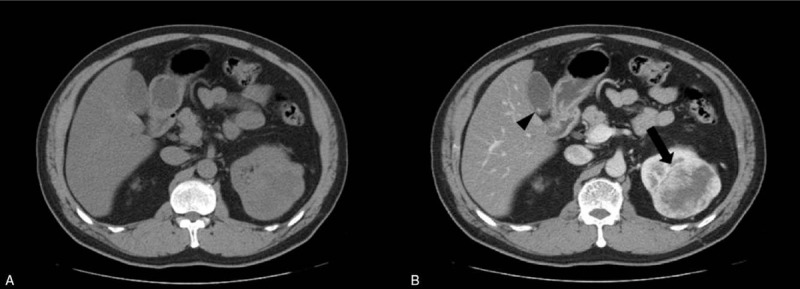
Computed tomography (CT) performed just before nephrectomy. (A) There are no perceptible gallbladder lesions in this unenhanced CT image. (B) Porta-phase axial CT shows a 9-cm tumor at the upper pole of the left kidney (arrow) and 5-mm enhancing gallbladder polyp (arrowhead).

In February 2019, a 55-year-old man was referred to our department for further evaluation and treatment of a gallbladder mass. He had a history of nephrectomy for clear-cell RCC of the left kidney. Contrast-enhanced computed tomography (CT) performed just before the nephrectomy revealed a tumor measuring 9 cm at the upper pole of the left kidney that exhibited exophytic growth and had a heterogeneous solid and cystic consistency. Simultaneously, a small enhancing polyp measuring 5 mm in diameter was identified at the liver side of the gallbladder body (Fig. 1). In December 2015, the patient had undergone an open radical nephrectomy, and the pathology report at that time revealed clear-cell RCC Fuhrman grade 3, and that pathological stage was T3 Nx.

**Figure 2:**
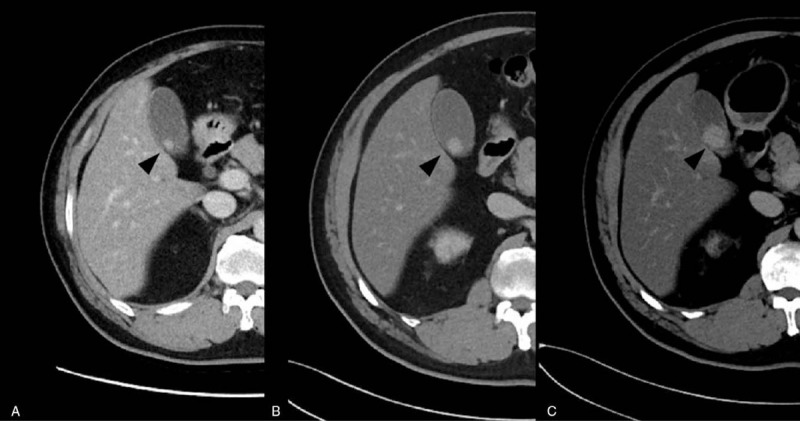
Computed tomography shows growth of the gallbladder polyp (arrowhead). (A) 1 yr after the nephrectomy. (B) 34 mo after the nephrectomy. (C) 38 mo after the nephrectomy.

**Figure 3:**
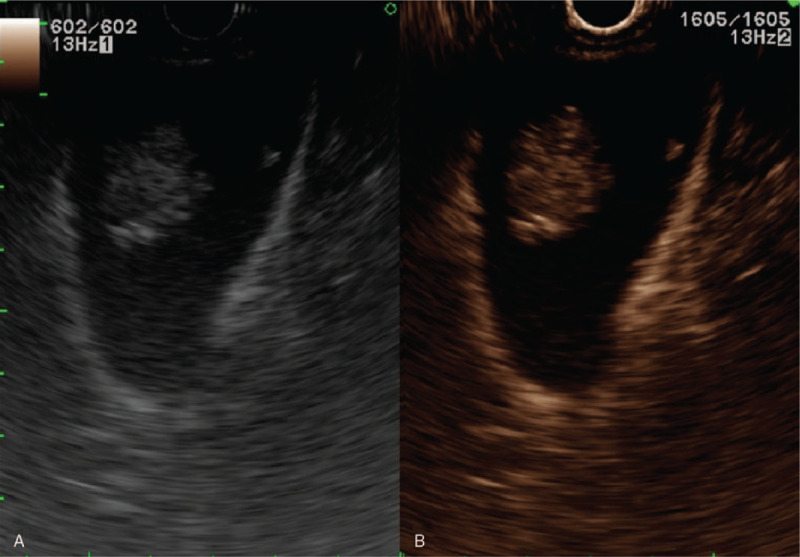
The gallbladder mass measures 24 mm and shows enhancement on contrast-enhanced ultrasonography (CEUS). (A) Gray-scale image. (B) CEUS image of the lesion.

The patient was followed up with CT scanning in an outpatient clinic, which indicated no suspicious lesions suggesting the recurrence of RCC except for the gallbladder polyp, whose diameter grew progressively by 5 mm in the following 34 months. The last CT scan performed before the patient's cholecystectomy in February 2019 showed that the polypoid gallbladder mass had grown and had a maximum diameter of 22 mm (Fig. 2). Endoscopic ultrasonography showed a 24 mm smooth-surfaced, heterogeneously echogenic, polypoid mass. After contrast injection with Sono-Vue (Bracco, Milan, Italy), the lesion was shown to be well enhanced and the gallbladder wall under the lesion was intact (Fig. 3). A chest CT and bone scan for evaluation of metastases were negative.

**Figure 4:**
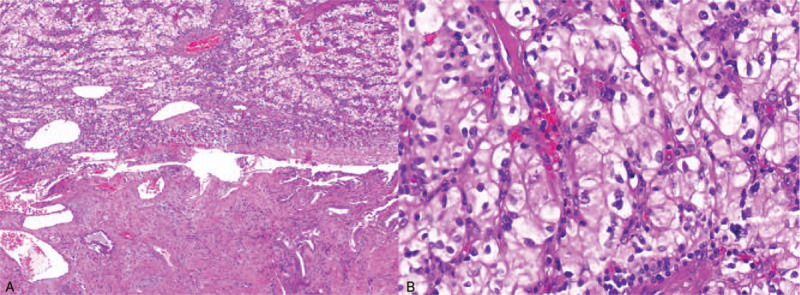
Hematoxylin and eosin staining. (A) The lesion contains clear cells confined to the polyp. (B) Image at 400× magnification shows abundant clear cytoplasm and round nuclei nested within a fibrovascular network.

We performed a laparoscopic cholecystectomy in April 2019. Gross examination of the specimen showed a pink–red polypoid mass measuring 23 mm in the largest dimension that was detached from the inner mucosal layer. To decide on the extent of surgery needed, we checked an intraoperative frozen biopsy, which showed an RCC metastasis without involvement of the muscular layer. The final pathology diagnosis was gallbladder metastasis of clear-cell RCC. Microscopically, the lesion comprised malignant clear cells that were confined to the polyp and a negative margin (Fig. 4). Immunohistochemistry was positive for CK and CD10, and Ki-67 expression was 20%.

To detect other possible metastases, we requested positron emission tomography after the cholecystectomy, which showed no lesions of the metastasis. The patient is free from disease and is in a satisfactory condition 10 months after the cholecystectomy. The patient provided written informed consent for publication of this report.

## 3 Discussion

In this case, a 5 mm gallbladder polyp showed enhancement on contrast CT and was identified at the body of the gallbladder before the nephrectomy. The polyp was confirmed as a synchronous solitary gallbladder metastasis of RCC 40 months after the nephrectomy.

Cholecystectomy for polypoid lesions of the gallbladder is traditionally performed according to the size threshold of 10 mm. However, in clinical practice, there is controversy whether cholecystectomy should be performed in patients with a gallbladder polyp smaller than 1 cm. Several factors should be considered when deciding whether to perform a cholecystectomy given the potential risk of malignancy; these factors include the patient's age and ethnicity; the lesion's morphology, size, and growth; and the presence and number of gallstones. A European group has recommended performing a cholecystectomy in patients with polyps measuring 6 to 9 mm if the patient has risk factors for gallbladder malignancy such as age >50 years, primary sclerosing cholangitis, Indian ethnicity, or a sessile polyp, and follow-up ultrasound for polyps <6 mm or measuring 6 to 9 mm in patients with no risk factors.^[6]^ Bhatt et al^[7]^ also reported that patients aged >50 years, sessile polyps, and single polyps are independent risk factors for malignancy; for polyps measuring 4 to 10 mm, they recommended cholecystectomy according to the risk probability of malignancy.

Despite the variety of imaging modalities available, it can be difficult to distinguish neoplastic polyps from nonneoplastic polyps because of their significant overlap in appearance. Transabdominal ultrasonography is good for detecting gallbladder polyps, but it has a poor discriminating ability between true and pseudo polyps.^[8]^ In a CT study, Choi et al^[9]^ analyzed the imaging features of gallbladder metastasis and reported that the characteristic pattern of gallbladder metastasis from RCC is the appearance of polypoid lesions with strong enhancement in the early and late arterial phase and wash-out in the portal phase. However, another study found no significant differences in enhancement pattern between nonneoplastic and neoplastic polyps.^[10]^ Moreover, for small polyps measuring <1 cm, imaging studies are limited in their ability to identify the characteristics associated with malignancy.

Given the difficulties mentioned above, deciding to perform a cholecystectomy at the time of initial diagnosis presented as a dilemma for the surgeons because the polyp measured <6 mm and it was not perceptible on an unenhanced CT image, which is a suggestive feature of cholesterol polyps.^[11]^ In other words, there was no definitive reason to perform the cholecystectomy according to the conventional indications.

However, there seems to have been a mistake in the management of the gallbladder polyp in this patient. The follow-up CT showed that the polyp had grown progressively to 10 mm 34 months after the initial presentation. Therefore, the cholecystectomy should have at least been performed at that time.

Distant metastasis from RCC usually have a poor outcome, and the 5-year survival rate is <10%. However, in contrast to typical metastases, solitary gallbladder metastasis of RCC have a more favorable patient survival. Shyr et al^[5]^ reported a 5-year survival rate of 80% in RCC patient with solitary gallbladder metastasis and concluded that gallbladder metastasis from RCC is not always at an advanced stage with a poor outcome. We also presume that a solitary gallbladder metastasis of RCC may exhibit different disease behaviors compared with typical metastases because no concomitant metastases occurred during the 40-month follow-up in this patient.

## 4 Conclusions

Solitary gallbladder metastasis of RCC may have a more favorable outcome than typical metastases. Although gallbladder metastasis of RCC is very rare, it can occur, and any changes in a gallbladder polyp in RCC patients should be managed under a strong suspicion of metastasis.

## Author contributions

**Conceptualization:** Sung Hoon Cho, Hyung Jun Kwon, Jae Min Chun.

**Resources:** Ja Ryung Han, Seock Hwan Choi, Hyun Tae Kim, Man-Hoon Han, Jae Min Chun.

**Supervision:** Young Seok Han.

**Writing – original draft:** Sung Hoon Cho.

**Writing –- review & editing:** Sung Hoon Cho, Young Seok Han, Jae Min Chun.
